# Angiotensin-2 receptors (AT1-R and AT2-R), new prognostic factors for renal clear-cell carcinoma?

**DOI:** 10.1038/sj.bjc.6605866

**Published:** 2010-11-23

**Authors:** T Dolley-Hitze, F Jouan, B Martin, S Mottier, J Edeline, O Moranne, P Le Pogamp, M-A Belaud-Rotureau, J-J Patard, N Rioux-Leclercq, C Vigneau

**Affiliations:** 1 CNRS UMR6061/IFR140, Faculté de Médecine Université de Rennes 1, 2 avenue du professeur Léon Bernard, CS34317, 35043 Rennes Cedex, France; 2 Service de Néphrologie, CHU Pontchaillou – 2 rue Henri Le Guilloux, 35033 Rennes Cedex, France; 3 Service d’Urologie, CHU Pontchaillou – 2 rue Henri Le Guilloux, 35033 Rennes Cedex, France; 4 Service d’Anatomie et Cytologie Pathologiques - CHU Pontchaillou – 2 rue Henri Le Guilloux, 35033 Rennes Cedex, France; 5 Service de Néphrologie, CHU Nice. Hôpital Pasteur, 2 voie Romaine, 06000 Nice, France

**Keywords:** renal cell carcinoma, AT1-R, AT2-R, Angiotensin-2, prognostic factor

## Abstract

**Background:**

The growth factor Angiotensin-2 signals through Angiotensin receptor type 1 (AT1-R) in a broad range of cell types and tumours and through the type-2 receptor (AT2-R) in a more restricted group of cell types. Although numerous forms of cancer have been shown to overexpress AT1-R, expression of AT1-R and AT2-R by human renal clear-cell carcinoma (RCCC) is not well understood. In this study, the expression of both angiotensin receptors was quantified in a retrospective series of RCCC and correlated with prognostic factors.

**Methods:**

Angiotensin receptor type 1 and AT2-R expressions were quantified on tumour tissues by immunohistochemistry (IHC), western blot and quantitative reverse transcriptase PCR (qRT–PCR). IHC results were correlated to Fuhrman's grade and patient progression-free survival (PFS).

**Results:**

A total of 84 RCCC were analysed. By IHC, AT1-R and AT2-R were expressed to a greater level in high-grade tumours (AT1-R: *P*<0.001, AT2-R: *P*<0.001). Univariate analysis showed a correlation between PFS and AT1-R or AT2-R expression (*P*=0.001). By multivariate analysis, only AT2-R expression correlated with PFS (HR 1.021, *P*=0.006) and cancer stage (*P*<0.001). By western blot, AT1-R and AT1-R were also found to be overexpressed in higher Fuhrman's grade (*P*<0.01 and *P*=0.001 respectively). By qRT–PCR, AT1-R but not AT2-R mRNA were downregulated (*P*=0.001 and *P*=0.118, respectively).

**Conclusion:**

Our results show that AT1-R and AT2-R proteins are overexpressed in the most aggressive forms of RCCC and that AT2-R expression correlates with PFS. AT1-R or AT2-R blockage could, therefore, offer novel directions for anti-RCCC therapy.

Renal clear-cell carcinoma (RCCC) accounts for 3% of adult cancers. Surgical resection is the mainstay of treatment, but about 40% of patients develop distant metastases with poor prognosis. The renin–angiotensin system (RAS) is well-known for its regulation of arterial pressure. RAS blockage represents an important target in the treatment of hypertension (HTA) and cardiac failure through two pharmacological classes: angiotensin-converting enzyme inhibitors (ACEi) and angiotensin-receptor blockers (ARB). The intracellular effects of angiotensin-2 (ATII) are principally mediated by two receptors: Angiotensin-2 receptor type 1 (AT1-R) and Angiotensin-2 Receptor type 2 (AT2-R).

Physiologically, both receptors are widely expressed, principally by heart, vessels, brain and kidney. Both receptors are involved during renal development (Norwood *et al*, 1997). In normal adult kidneys, they localise in the cortex where AT1-R mRNA is expressed 8–10 fold more than AT2-R mRNA (Matsubara *et al*, 1998). Angiotensin receptor type 1 is strongly expressed by mesangial cells (Goldfarb *et al*, 1994; Arendshorst *et al*, 1999) and interlobular endothelial arterial cells (Matsubara *et al*, 1998; Arendshorst *et al*, 1999). In contrast, AT2-R is expressed more strongly by pre-glomerular endothelial arterial cells and by interlobular endothelial arterial cells (Goldfarb *et al*, 1994; Matsubara *et al*, 1998; Arendshorst *et al*, 1999). Both receptors are also expressed by proximal tubular cells (Weerackody *et al*, 1997).

Angiotensin-2 receptors regulate many physiological processes. The main function of ATII is vasoconstriction, as one of the final steps of RAS (Arendshorst *et al*, 1999). In many cells, ATII, through AT1-R facilitates cellular proliferation and angiogenesis (Stoll *et al*, 1995; Li *et al*, 2008), whereas AT2-R has anti-proliferative properties (Stoll *et al*, 1995; Nouet and Nahmias, 2000). Moreover in the kidney, ATII induced tubular cell hypertrophia and proliferation through AT1-R (Chatterjee *et al*, 1997). In addition, in the kidney, ATII, through AT2-R, triggered tubular cell proliferation, angiogenesis and apoptosis (Cao *et al*, 2000). As RCCC cells are developed from tubular renal cells, AT1-R and AT2-R may also be involved in RCCC development.

Recently, overexpression of AT1-R by *in vitro* cultured breast-carcinoma cells (Herr *et al*, 2008; Rhodes *et al*, 2009), pancreatic adenocarcinoma cells (Arafat *et al*, 2007) or hepatocarcinoma cells (Yoshiji *et al*, 2002) was reported. Overexpression of AT1-R has also been demonstrated *in vivo* in different tumours, including: oestrogen receptor positive breast cancers (Rhodes *et al*, 2009), glioblastomas (Juillerat-Jeanneret *et al*, 2004), ovarian cancers (Suganuma *et al*, 2005), squamous cell carcinomas of the skin (Takeda and Kondo, 2001) and gastric cancers (Rocken *et al*, 2007). Despite its anti-proliferative effect, AT2-R was also found to be overexpressed by several cancers such as astrocytomas (Arrieta *et al*, 2008) or lung tumours (Tamura *et al*, 2008) *in vivo*.

Several clinical series examined the link between AT1-R or AT2-R overexpression and patient prognosis. Angiotensin receptor type 1 and AT2-R overexpression was linked to worse prognosis (Arrieta *et al*, 2008) in astrocytomas, suggesting a role for these receptors on carcinogenesis and/or neoangiogenesis.

Little is known about the expression and role(s) of ATII receptors in RCCC. An initial study described expression in RCCC by autoradiography but did not correlate this expression with tumour aggressiveness (Goldfarb *et al*, 1994). A second study showed on a mouse model that ARB could prevent the development of RCCC pulmonary metastasis (Miyajima *et al*, 2002). The objective of our study was to quantify the expression of both ATII receptors in RCCC and correlate this expression with well-known prognostic factors and progression-free survival (PFS).

## Materials and methods

This study was designed retrospectively by using the Rennes database for RCCC from 2002 to 2006 and by exploring expression of angiotensin-2 receptors by several methods. All patients undergoing nephrectomy for sporadic RCCC in Rennes are included in a clinical and histological database, after giving informed consent.

### Tumours specimens

Immediately after macroscopic examination, small samples are collected from surgical specimens, frozen in liquid nitrogen and stored at −80°C for RNA and protein extraction. Tumour tissues were also preserved in 10% formalin and embedded in paraffin for immunostaining. For protein or RNA extractions, 5 *μ*m tissue sections were cut before and after the samples. Hemotoxylin–eosin staining was performed on sections to confirm or revise the Fuhrman's grade and only controlled samples were included. If necrosis, fibrosis or non-tumourous kidney tissue was above 10% of total tissue examined, the corresponding sample was excluded from analysis.

### Pathological, histological and clinical data

In this study, TNM (Tumour, Node and Metastasis) score, tumour size and Fuhrman's grade were extracted from Rennes database for RCCC. These data are routinely evaluated for each tumour. Fuhrman's grade, a nuclear grading system, is based on nuclear size, shape and prominence of nucleoli. This score is determined in the most aggressive tumour area and represents one of the key determinants of RCCC-specific survival (Fuhrman *et al*, 1982). Several clinical data were also used such as gender, age, delay between surgery and progression or death and performance status at surgical time (ECOG scale (Eastern Cooperative Oncology Group) (Oken *et al*, 1982)).

### Immunohistochemistry (IHC)

For immunostaining, a representative paraffin block with the highest Furhman's grade was selected; 5 *μ*m-thick sections were cut from each block. Immunostaining was performed on formalin-fixed, paraffin-embedded tissues using the labelled-polymer method. Slides were dewaxed and rehydrated using xylol and ethanol, respectively, and transferred in hot citrate buffer 0.01 mol l^−1^ and pH 6 (cooked in 100°C, 40 min), left in the hot buffer for 20 additional minutes and transferred into PBS-T 0.1% buffer for 5 min. Endogenous peroxidase was quenched in 3% hydrogen peroxide for 10 min and slides were again transferred into PBS-Tween 0.1% buffer for 5 min. After pre-incubation with FCS (fetal calf serum) 10% for 20 min (for AT1-R), the slides were incubated with a dilution of 1:500 (in PBS-T 1% FCS) rabbit polyclonal anti AT1-R antibody (Santa-Cruz, NT, USA; sc-1173) and a dilution of 1:100 (in primary antibody-diluting buffer) goat polyclonal anti AT2-R Antibody (Santa-Cruz; K15, sc-48452) at room temperature for 2 h. Control without primary antibody was always performed at the same time with PBS-T 1% SVF (AT1-R), and primary antibody-diluting buffer (AT2-R). Sections were rinsed in PBS-T and incubated with polyclonal rabbit anti-goat immunoglobulin (1:200 dilution-30 min) for AT2-R only, followed by incubation with HRP (horseradish peroxidase)-labelled polymer conjugated with secondary antibodies (Envision + Dual link system-HRP, DAKO). Diaminobenzidine was used as a chromogen in the presence of hydrogen peroxide. Hematoxylin was used as a couterstainer. Study specimens were evaluated by a single uropathologist (NRL) (Microscope: olympus BX51, camera: olympus DP70). Immunostaining of AT1-R and AT2-R was expressed as the percentage of AT1-R/AT2-R-positive tumour cells by scoring at least 1000 cells.

### Western blot analysis

Protein lysates were prepared by treating frozen tissues with RIPA (radio immuno precipitation assay) buffer (consisting of 50 mM Tris-HCL pH8, 150 mM NaCl, 0,1% SDS and 1% NP40, 0,5% Na deoxycholate, 1 mM DTT and 10 *μ*g ml^−1^ protease inhibitors) at 4°C (30–60 min). Tissue lysates were then centrifugated at 13 000 g for 30 min and supernatants were harvested. Protein concentrations were determined by the Bradford method. A volume of 25 *μ*g of protein (boiled at 95°C) were separated by 12.5% SDS–polyacrylamide gel electrophoresis (SDS–PAGE) and transferred to a nitrocellulose membrane. Each membrane was blocked with phosphate buffer saline (PBS) containing 5% skim milk for 2 h, and incubated at 4°C overnight with 1:500 of AT1-R (Santa-Cruz; sc-1173) or 1:200 of AT2-R (Santa-Cruz; H 143, sc-9040). The membrane was subsequently incubated for 1 h at room temperature with fluorescent-labeled secondary antibody (IRDye Tmn 800-dilution 1:5000) and analysed using infrared imaging system (Odyssey-Biosciences). *β*-Actin was used as loading and blotting controls and detected by anti-human *β*-actin (clone AC-15, Sigma, Lyon, France).

### RNA (ribonucleic acid) purification and quantitative RT–PCR (reverse transcript PCR)

Total RNA was extracted from tissues with the Genelute Mammalian total RNA Miniprep (RTN-70 Sigma), and treated with DNase I to avoid genomic DNA contamination. The RNA product was quantified by absorbance at 260 nm. The integrity of RNA samples was controlled by analysis on bioanalyzer with RNA 6000 Nano assay Labship (Agilent Technologies, Santa Clara, CA, USA) and ratio integral number (RIN) was calculated. Only RNA with RIN >8 were further processed and used for cDNA synthesis. A volume of 1 *μ*g of total RNA was transcribed into cDNA using high capacity cDNA Archive kit (Applied Biosystems, Foster City, CA, USA).

Quantitative PCR was performed with the PCRq 7900 HT (Applied biosystems) using power SYBR Green PCR Master mix (Applied biosystems). The reaction mixture contained 5 *μ*l of master mix, 0.3 *μ*l of each primer (10 *μ*M) and 2 *μ*l cDNA (diluted 1:40) for a total reaction volume of 10 *μ*l. Oligonucleotide primers were synthesised by Eurogentec (Angers, France). The sequences of these primers were as follows: (forward) 5′-ATG-ATT-CCA-GCG-CCT-GAC-3′ and (reverse) 5′-GGT-CCA-GAC-GTC-CTG-TCA-CT-3′ for AT1-R, (forward) 5′-GGT-TTC-TAG-CAT-ATA-CAT-CTT-CAA-CCT-3′ and (reverse) 5′-GCC-CAT-AGA-GGA-AGA-GTA-GCC-3′ for AT2-R, (forward) 5′-GGT-CCT-TTC-ACC-AGC-AAG-CT-3′ and (reverse) 5′-GCT-TTC-CTT-GGT-CAG-GCA-GTA-3′ for HPRT. The programme was as follows: an initial step at 95°C for 10 mn and then 40 cycles of 95°C for 15 s and 60°C for 60 s. Regression curves were drawn for each sample, and its relative amount was calculated from the threshold with the instrument's software (SDS 2.0) according to the manufacturer's instructions. Relative expression levels of the target genes were normalised mean of an internal control gene, HPRT.

### Statistical analysis

Progression-free survival was determined from the date of surgery to the date of cancer progression or the last follow-up. Non-parametric tests (Kruskal Wallis) were used to compare means and proportions. Estimates of the cumulative survival distributions were calculated according to the Kaplan–Meier method and log-rank tests were used to compare the differences between groups. A Cox proportional-hazard regression model was used to test the independent effects of clinical and pathological variables on survival. Graphical methods suggested that the proportional-hazards assumption was reasonable for all selected variables. A stepwise selection procedure was used to select the final optimal model. All *P*-values were two-sided and *P*<0.05 was considered to indicate significance. All analyses were conducted with the Statistical Package for the Social Sciences, version 16.0 (SPSS Inc., Chicago, IL, USA).

## Results

A total of 84 patients who underwent nephrectomy between 2002 and 2006 were included in our study. All protein and RNA extracts from frozen samples corresponding to IHC Fuhrman's grade were selected for western blot and qRT–PCR experiments (*n*=55). Paraffin sections were done on all samples.

In all, 50 men and 34 women were included, with a mean age of 63.7±11.3 years. At the time of surgery, 56 patients were ECOG 0 and 28 ECOG 1; three tumours were Fuhrman's grade 1 (3.5%), 32 grade 2 (37.6%), 26 grade 3 (30.9%) and 23 grade 4 (27.3%). A total of 22 patients (26.2%) had metastases at the time of surgery, as well as 11 (13.1%) had lymph node involvement. Median tumour size was 7.0±4.10 cm. No patient received anti-angiogenic therapy or chemotherapy before surgery. Mean follow-up was 30 months (±18 months).

For the statistical analysis grade 1 and 2 were combined because of low number of grade 1 tumours.

### Immunohistochemistry

**Figure 1:**
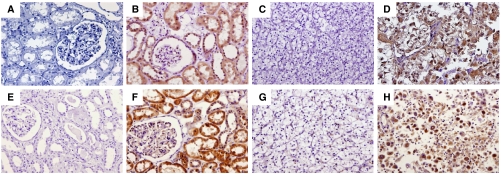
Staining by IHC on non-tumorous kidney tissue and renal cell carcinoma. Top panel, AT1-R IHC: (**A**) negative control, (**B**) staining of non-tumorous renal tissue, positive on tubular and Bowman's capsule cells, (**C**) Fuhrman's grade 1 renal carcinoma with no staining, (**D**) Fuhrman's grade 4 renal carcinoma with positive staining. Bottom panel, AT2-R IHC: (**E**) negative control, (**F**) staining of non-tumorous renal tissue on tubular cells, (**G**) Fuhrman's grade 1 renal carcinoma with no staining, (**H**) Fuhrman's grade 4 renal carcinoma with intense staining. × 200 magnification for all images.

Localisation of AT1-R and AT2-R was described on tumour tissue and normal surrounding tissue (Figure 1). Angiotensin receptor type 1 stained all tubular cells, podocytes, epithelial cells of Bowman's capsule and vascular smooth-muscle cells in the normal cortex. AT2-R cortical staining localised only on tubular cells. Tumour cells, as well as normal renal cells, exhibited cytoplasmic staining but not membrane or nuclear immunostaining.

**Figure 2:**
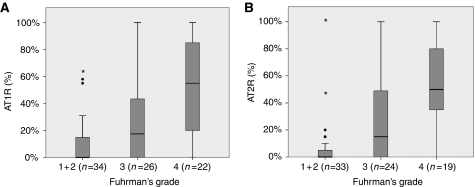
Angiotensin receptor type 1 (**A**) and AT2-R (**B**) expression by IHC according to Fuhrman's grade. Results are expressed as percentage of AT1-R/AT2-R-positive tumour cells. Median expression is 12.5% for AT1-R and 10% for AT2-R. Angiotensin receptor type 1 (*n*=82 tumours) and AT2-R (*n*=76 tumours) are overexpressed by the most aggressive tumours (*P*<0.001 for both).

A total of 82 tumours were stained for AT1-R (3 Fuhrman's grade 1, 31 grade 2, 26 grade 3 and 22 grade 4) and 76 for AT2-R (3 Fuhrman's grade 1, 30 Fuhrman's grade 2, 24 Fuhrman's grade 3 and 19 Fuhrman's grade 4). For each sample, the ratio of positive tumour cells/total tumour cells was expressed as a percentage. Angiotensin receptor type 1 median ratio was 12.5 % and AT2-R one was 10%. Figure 2 shows that AT1-R was overexpressed by Fuhrman's 4 RCCC compared with other grade tumours (*P*<0.001; panel A). Angiotensin type-2 receptor was also overexpressed by Fuhrman's 4 RCCC (*P*<0.001; panel B).

### Western blot

**Figure 3:**
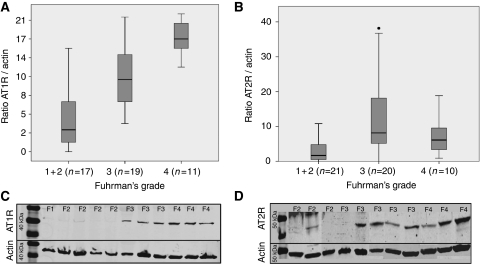
Quantification by western blot of AT1-R/Actin ratio (**A**, **C**) and AT2-R/Actin ratio (**B**, **D**). Angiotensin receptor type 1 is over expressed by the higher Fuhrman's grades tumours (*P*<0.001, *n*=47), as well as AT2-R (*P*=0.001, *n*=51). F for Fuhrman grade.

Frozen tumours were included in the WB experiments to confirm IHC results by another semi-quantitative method (Figure 3). A total of 47 frozen samples (2 Fuhrman's grade 1, 15 grade 2, 19 grade 3 and 11 grade 4) containing tumour tissue alone were analysed. Samples with significant proportion of non-tumour tissue or fibrosis or necrosis (more than 10%) were excluded. The AT1-R was overexpressed by the higher Fuhrman's grade tumours as shown in Figure 3 (*P*<0.001); panel A). A total of 51 samples were included to quantify AT2-R by WB (3 Fuhrman's grade 1, 18 grade 2, 20 grade 3 and 10 grade 4). Angiotensin type-2 receptor was also significantly increased in high Fuhrman's grade tumours (*P*=0.001; panel B).

### qRT–PCR

**Figure 4:**
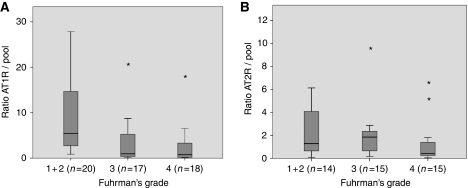
Reverse transcription–PCR of AT1-R and AT2-R according to Fuhrman's grade. Angiotensin receptor type 1 expression is significantly decreased according to the Fuhrman's grade (panel **A**, *P*=0.001, *n*=55). Angiotensin type 2 receptor is not differentially expressed between different Fuhrman's grades (panel **B**; *P*=0.118, *n*=44).

By RT–PCR, AT1-R expression was analysed on 55 samples (3 grade 1 tumours, 17 grade 2, 17 grade 3 and 18 grade 4). It was significantly decreased according to the Fuhrman's grade (Figure 4 - panel A, *P*<0.001). However, on the 44 tumours suitable for analysis (2 grade 1, 12 grade 2, 15 grade 3 and 15 grade 4), AT2-R mRNA was not differentially expressed according to Fuhrman's grade (panel B; *P*=0.118, NS).

### Survival, univariate and multivariate analyses

**Figure 5:**
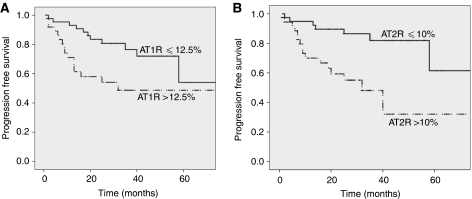
Patient progression-free survival curves according to AT1-R (**A**) and AT2-R (**B**) level, assessed by IHC. (**A**) AT1-R⩽median (plain line) or >median (dash) *P*=0.006; (**B**) AT1-R⩽median (plain line) or >median (dash) *P*=0.001 (log-rank test).

Progression-free survival was assessed by the Kaplan–Meier method and compared by log-rank test according to the level of AT1-R and AT2-R (⩽ or >median expression determined by IHC method). Figure 5 shows that PFS was better when AT1-R was expressed below 12.5 % (median) by IHC (*P*=0.006; panel A) and AT2-R below 10% (median) (*P*=0.001; panel B). Univariate and multivariate analysis results are presented in Table 1 for AT1-R and in Table 2 for AT2-R. By univariate analysis, PFS correlated with AT1-R (*P*=0.001), as well as stage (T1/2 vs T3/4, *P*<0.001), nodal invasion (*P*<0.001), distant metastasis (*P*<0.001), tumour size (⩽ or >7 cm, *P*=0.001), Fuhrman's grade (F1/2 vs F3/4 *P*<0.001) but not ECOG stage (*P*=0.239). By multivariate analysis, AT1-R did not correlate with PFS (NS) on the contrary to tumour stage (*P*<0.001), Fuhrman's grade (*P*=0.044), nodal invasion (*P*=0.003) and ECOG stage (*P*=0.005). For AT2-R, univariate analysis showed that PFS correlated with AT2-R (*P*=0.001), as well as tumour stage (T1/2 vs T3/4, *P*<0.001), nodal invasion (*P*=0.002), distant metastasis (*P*<0.001), tumour size (< or >7 cm, *P*=0.001), Fuhrman's grade (F1/2 vs F3/4 *P*<0.001) but not ECOG stage (0.253). By multivariate analysis, AT2-R was still correlated with PFS (*P*=0.006) as tumour stage (*P*<0.001).

## Discussion

Our study shows that both AT1-R and AT2-R protein expression correlate with RCCC aggressiveness and PFS. This relationship suggests a role for these two receptors in regulating tumour growth and/or neoangiogenesis and a potential therapeutic effect of receptor antagonists.

AT1-R overexpression has already been described in other cancers but not previously for RCCC. This correlation might be because of a direct effect of ATII on tumour cells. Indeed, ATII through AT1-R can activate tumour cell proliferation through at least two simultaneous mechanisms: PI3-kinase/Akt pathway (Zhao *et al*, 2010) and EGFR (epithelial growth factor receptor) trans-activation (Fujiyama *et al*, 2001; Greco *et al*, 2003; Uemura *et al*, 2003). Secondly, ATII through AT1-R can induce an increased expression and production in vascular endothelial growth factor (VEGF) inducing neo-angiogenesis (Arafat *et al*, 2007; Kosaka *et al*, 2010). Induction might be triggered by stabilisation of hypoxia inducible factor-1*α* (HIF1α), as shown in prostate cancer model (Richard *et al*, 2000; Ino *et al*, 2006; Kosaka *et al*, 2010). Positive correlation between AT1-R expression and VEGF production has been showed in ovarian cancer, *in vivo*. Moreover, ATII through AT1-R reduces tumour cell adhesion although reducing invasion of the basement membrane (Puddefoot *et al*, 2006). Lastly, in a mouse model of gastric cancer AT1-R could trigger lymphangiogenesis (Wang *et al*, 2008).

AT2-R is overexpressed by some cancers such as gastric cancers (Rocken *et al*, 2007) though its role in carcinogenesis remains controversial. Under physiological conditions, AT2-R has anti-proliferative properties (Fujiyama *et al*, 2001), whereas during carcinogenesis, AT2-R can induce an invasive phenotype, for example, in gastric cancer (Carl-McGrath *et al*, 2007), or prevent cell proliferation (Bose *et al*, 2009). However, AT2-R has been shown to counteract the effects of ATII mediated by AT1-R, and might be upregulated upon prolonged ATII stimulation (Nouet and Nahmias, 2000). High expression of AT2-R in RCCC could be either a consequence of AT1-R overexpression or of the high circulating level of ATII and/or may have specific angiogenesis effects as in gastric cancer. Even more, in kidney, AT2-R can physiologically induce tubular cell proliferation during development or after kidney injury (Cao *et al*, 2000). As RCCC develops from tubular cells, AT2-R overexpression may be one of the mechanisms by which tubular cell proliferation develops into carcinogenesis. The strong correlation between tumour aggressiveness, PFS and AT2-R expression in our study indeed supports the hypothesis for a proliferative effect of AT2-R in RCCC, more than in other type of cancers. Specific *in vitro* studies, using anti AT1-R and/or AT2-R inhibitors on RCCC-cultured cells will help to illuminate the role(s) of AT2-R in renal carcinogenesis.

In our *ex vivo* study, dissociation between AT1-R and AT2-R protein expression and RNA synthesis was also observed. As the staining of both receptors was mainly expressed by tumour cells as detected by IHC, and because the tumour nature of tissue analysed was controlled, we suggest that AT1-R and AT2-R mRNA amplified were indeed synthesised by tumour cells. We also suggest that post-transcriptional or post-translational mechanisms, never described in other type of cancer, might be involved in RCCC. The underlying mechanisms of receptor synthesis, internalisation, sequestration and inactivation are incompletely understood (Arendshorst *et al*, 1999).

The correlation between RAS and RCCC opens new potential therapeutic strategies in RCCC. Angiotensin-receptor blockers and ACEi are highly prescribed and well tolerated anti-hypertensive therapies. Large epidemiological studies suggest potential protective effects against cancer risk even if results remain controversial (Lever *et al*, 1998; Friis *et al*, 2001). Angiotensin-receptor blockers and ACEi could act on tumour progression by two different mechanisms: inhibition of cancer proliferation and/or inhibition of neovascularization. In experimental studies on different cancer types, ARB or ACEi showed anti-proliferative effects: in breast cancer cells (Puddefoot *et al*, 2006; Rhodes *et al*, 2009) in human melanoma xenograft model (de Groot-Besseling *et al*, 2004), in murine hepatocellular carcinoma (Noguchi *et al*, 2003), in colorectal cancer liver metastases (Neo *et al*, 2007), on pancreatic cancer cells (Arafat *et al*, 2007) or on lung metastases of mouse RCCC (Miyajima *et al*, 2002). Inhibition of tumour growth involves the decreased expression of VEGF. It has been shown for pancreatic cancer (Arafat *et al*, 2007; Noguchi *et al*, 2009) and for prostate cancer (Kosaka *et al*, 2010). The inhibition of endothelial cell tubular formation (Yoshiji *et al*, 2005) and/or apoptosis of endothelial or tumour cells (De la Iglesia Inigo *et al*, 2009) were also described. In a recent model of murine melanoma graft, ARB-induced tumour growth decrease by low microvascular density, and the decrease of some angiogenic mediators such as VEGF-1 and VEGF-2 (Otake *et al*, 2009). In addition, in animal models, ARB or ACEi enhance the effect of usual chemotherapies: in murine hepatocarcinoma in association to interferon- (Noguchi *et al*, 2003), in mouse bladder cancer combined with cis-platin by further suppressing angiogenesis (Kosugi *et al*, 2009), by enhancing effects of radiation (Ohnuma *et al*, 2009), or in addition with K2 vitamin in hepatocellular carcinoma in rats (Yoshiji *et al*, 2006). Furthermore, few recent clinical trials reported that ARB could improve cancer prognosis when used in association with standard chemotherapies. In a report of three cases of metastatic RCCC, ARB was associated with cimetidine and cyclo-oxygenase 2 inhibitors allowing prolonged partial remission (Tatokoro *et al*, 2008). In a retrospective cohort study on advanced non-small-cell lung cancer, ARB or ACEi were statistically associated with longer patient survival when used with first-line platinum-based chemotherapy (Wilop *et al*, 2009).

One pharmacological AT2-R blocker (PD123319) has been used with controversial results. It can facilitate tumourogenesis by Pax2 activation and resulted in prostatic cancer-cell proliferation (Bose *et al*, 2009). In a gastric cancer model, the same AT2-R blocker induced cell proliferation and cell viability, but inhibited cell invasion (Carl-McGrath *et al*, 2007). In recent work on pancreatic cancer cells, it also enhanced production of the inflammatory mediator MCP-1, which promotes tumour invasion (Chehl *et al*, 2009). According to this tissue specificity of AT2-R expression and AT2-R blocker effect, overexpression of AT2-R in the RCCC may suggest a different role of AT2-R and in consequence, different actions of its blocker.

For RCCC, 40% of the patients develop distant metastases with poor prognosis. As RCCC are highly vascularises cancers, with high secretion of VEGF (Rioux-Leclercq *et al*, 2007), recent therapies are focused on anti-angiogenic drugs (Rini, 2009): anti-VEGF antibody (bevacizumab) (Rini *et al*, 2008) or tyrosine kinase inhibitors (Sunitinib, sorafenib) (Motzer *et al*, 2007; Negrier *et al*, 2009). These drugs already significantly increased patient survival but PFS is still counted in months (Motzer *et al*, 2007). Our study demonstrates that AT1-R and AT2-R are overexpressed in RCCC, and that this overexpression correlates with tumour aggressiveness and PFS. In consequence, AT1-R and AT2-R can be used as new prognosis factors for RCCC but ARB and ACEi can also be suggested as new adjuvant pathway for RCCC therapy. Indeed, these results, in addition to already-published experimental and clinical data on other cancer types, suggest that blocking RAS might be a way to decrease RCCC growth, neovascularization and metastasis dissemination. Experimental *in vitro* and *in vivo* studies with ARB and ACEi, in addition to antiangiogenic therapies, are the following steps to confirm our hypothesis.

## Conclusion

Our study first report that AT1-R and AT2-R protein expression can be used as new prognosis factors in RCCC, strongly related to tumour aggressiveness and worse PFS. These results support the hypothesis that ARB and ACEi could represent interesting adjuvant treatments in metastatic RCCC.

## Figures and Tables

**Table 1:** Variables associated with progression-free survival in univariate analysis and with Cox proportional hazards regression model for AT1-R expressed by IHC

**Variables (No. of patients)**	**Percentage of progression**	***P* (univariate analysis)**	**Multivarate analysis hazard ratios (95% CI)**	***P***
*Stage*				
T1/2	(45) 11.1 %			
T3/4	(39) 61.5 %	<0.001	9.841	<0.001
				
*Nodal invasion*				
No	(73) 28.7 %			
Yes	(11) 72.7 %	<0.001	4.245	0.003
				
*Distant metastasis*				
No	(47) 22.6 %			
Yes	(37) 68.2 %	<0.001		NS
				
*Tumor size*		0.001		NS
⩽7 cm	(44) 15.9 %			
>7 cm	(40) 55 %			
				
*Fuhrman's grade*				
1+2	(35) 8.5 %			
3+4	(49) 53.0 %	<0.001	3.688	0.044
				
*AT1R⩽ or >12.5%*				
⩽12.5%	(41) 25 %			
>12.5 %	(43) 44.7 %	0.001		NS
				
*ECOG*				
0	(56) 28.5 %			
1 or more	(28) 46.4 %	0.239	3.487	0.005

Abbreviations: AT1R=angiotensin type 1 receptor; CI=confidence interval; ECOG=eastern cooperative oncology group performance status.

**Table 2:** Variables associated with progression-free survival in univariate analysis and with Cox proportional hazards regression model for AT2-R expressed by IHC

**Variables (No. of patients)**	**Percentage of progression**	***P* (univariate analysis)**	**Multivarate analysis Hazard ratios (95% CI)**	***P***
*Stage*				
T1/2	(45) 11.1 %			
T3/4	(39) 61.5 %	<0.001	14.297	<0.001
				
*Nodal invasion*				
No	(73) 28.7 %			
Yes	(11) 72.7 %	0.002		NS
				
*Distant metastasis*				
No	(47) 22.6 %			
Yes	(37) 68.2 %	<0.001		NS
				
*Tumor size*		0.001		NS
⩽7 cm	(44) 15.9 %			
>7 cm	(40) 55 %			
				
*Fuhrman's grade*				
1+2	(35) 8.5 %			
3+4	(49) 53.0 %	<0.001		NS
				
*AT2R⩽ or >10 %*				
⩽10%	(42) 17.5 %			
>10%	(42) 47.2 %	0.001	1.021	0.006
				
*ECOG*				
0	(56) 28.5 %			
1 or more	(28) 46.4 %	0.253		NS

Abbreviations: AT1R=angiotensin type 1 receptor; CI=confidence interval; ECOG=eastern cooperative oncology group performance status.
